# Editorial: From canvas to care: artistic interventions as multimodal therapeutics in child and adolescent mental health

**DOI:** 10.3389/frhs.2026.2013940

**Published:** 2026-10-14

**Authors:** Weifeng Han, Ben Wadham, Katherine M. Boydell, Deb Agnew, Amy Hamilton

**Affiliations:** 1 Charles Darwin University, Darwin, NT, Australia; 2 Flinders University, Adelaide, SA, Australia; 3 Black Dog Institute, Department of Psychiatry and Mental Health, University of New South Wales, Randwick, NSW, Australia

**Keywords:** arts-based interventions, child and adolescent mental health, creative expression, mental health services, school-based mental health

When we launched this Research Topic, we started from a simple premise: child and adolescent mental health care should not depend only on spoken language. Children and young people may have limited, developing, culturally shaped, or disrupted ways of communicating. Communication difficulties can also intersect with emotional and behavioural concerns, which oftentimes makes it harder for some young people to engage with services that rely heavily on talk (1). We, therefore, invited work across mental health, education, speech-language pathology, expressive arts, and child development. The aim was not to promote art as a standalone form of mental health support. Instead, we asked how visual art, music, movement, drama, play, and other creative forms might support wellbeing, emotional regulation, communication, and participation across clinical, school, and community settings.

The nine articles in this Research Topic give a useful but cautious answer. Vasilopoulos et al. reviewed 34 studies of arts-inclusive programs for children aged 6 to 12 years. Some structured programs showed promising results for stress, wellbeing, and general mental health, but the certainty of evidence was low or very low for most outcomes. The review also identified possible psychological, biological, and social pathways, but none of the included studies formally tested whether these processes caused changes in outcomes. Jankelowitz et al. reached similar conclusions in their review of sand-based therapy. Traditional sand therapy showed promise, but the evidence had important design limits. The authors also found no empirical studies of digital sand therapy in children. These two reviews set an important boundary for the Topic. Creative practice is expanding faster than the evidence needed to guide its use.

At the same time, several articles show why creative approaches are important despite evidence of positive changes in clinical symptoms. Birnkammer et al., for example, found that therapists in Germany often used creative techniques to support emotional expression, engagement, communication, and access to difficult experiences. These techniques were usually associated with one diagnosis. Piché et al. found that children in an after-school arts program valued a safe and supportive environment built through positive relationships with peers, facilitators, and program staff. Their accounts also pointed to autonomy, competence, and relatedness. Gilbert et al. reported similar themes in a school-based program that combined art and philosophical inquiry. Children described connection, collaboration, empathy, respect, and self-awareness. Across these studies, art does not simply sit beside care as an activity. It can offer another way into communication, reflection, and relationships when direct verbal discussion is difficult or not enough.

The collection also places creative approaches across a wider care continuum. Grummitt et al. evaluated a whole-school partnership that combined creative expressive therapy for children affected by trauma with support for school staff. Students showed improved wellbeing over the program, while teachers reported greater knowledge of trauma-informed practice. Staffing, cultural differences, and long-term sustainability remained concerns. Jacob et al. examined a structured arts-based program for pupils with intellectual disability in a resource-constrained setting. Their small quasi-experimental study found improved attention after the intervention, therefore, suggesting that creative approaches may also support participation and learning. Law extends the school focus with a proposed model that combines artmaking and human dignity education to support student and teacher wellbeing. Golden et al., in turn, place arts-based activity within the broader youth mental health service landscape. Their research highlights unequal access, the role of schools and communities, and the need to involve young people in the design of support.

Equity is, therefore, not a side issue. Piché et al. focus on children from low-income and rural communities. Jacob et al. examine a low-resource educational setting. Golden et al. highlight gaps in access and the need for culturally responsive services. Birnkammer et al. show that creative methods may help therapists work with racially minoritised young people, while also identifying clear gaps in racism-informed training. Creative approaches are not culturally neutral simply because they rely less on speech. Their value depends on trust, cultural knowledge, practitioner skill, and the experience of the young people involved.

This is where the health service question becomes central. Moving from canvas to care requires more than showing that an activity is engaging or promising. Services need to determine who should receive these approaches, who should deliver them, what training is needed, how they connect with existing care, and how they can be sustained. Researchers also need to test longer-term outcomes, possible mechanisms, costs, access, and implementation across different cultural and service settings. Young people and families should have a stronger role in those decisions, and be meaningfully involved in all stages of the research from conception to dissemination. The next phase of this field should therefore move beyond exploring whether the arts support mental health and instead ask for whom they help, through which processes, in which settings, at what level of need, and under what service conditions. That shift is essential if creative practice is to become not only expressive, but also provides credible, equitable, and sustainable care.
